# Unmasking Ketosis-Prone Diabetes in a Young Adult

**DOI:** 10.7759/cureus.113179

**Published:** 2026-07-22

**Authors:** Angela E Gallucci, Victoria Powell, Franklin Thelmo

**Affiliations:** 1 Internal Medicine, ChristianaCare Health System, Newark, USA; 2 Internal Medicine-Pediatrics, ChristianaCare Health System, Newark, USA; 3 Endocrinology, Diabetes, and Metabolism, ChristianaCare Health System, Newark, USA

**Keywords:** diabetes, hyperglycemia, ketoacidosis, ketosis-prone diabetes, β-cell function

## Abstract

Ketosis-prone diabetes (KPD) is a unique clinical entity that does not fit the classic phenotypes of type 1 diabetes mellitus (T1DM) or type 2 diabetes mellitus (T2DM), although it can present similarly with severe hyperglycemia or diabetic ketoacidosis (DKA). This report describes a patient initially diagnosed with type 2 diabetes mellitus who was subsequently suspected to have ketosis-prone diabetes based on his clinical course.

A 22-year-old man presented to the emergency department (ED) with hyperglycemia and was diagnosed with new-onset type 2 diabetes. He was closely monitored by his primary care physician (PCP) and outpatient pharmacist but continued to experience hyperglycemia. He was prescribed increasing doses of insulin, reaching up to 56 units of basal insulin nightly and 12 units of prandial insulin. Antibody testing was mildly elevated, although determined to be clinically insignificant. The patient utilized continuous glucose monitoring (CGM), and after several months of insulin therapy, he experienced hypoglycemic episodes. Consequently, he was titrated off all insulin therapy and successfully maintained euglycemia for eight months before experiencing disease relapse. Ketosis-prone diabetes is challenging to diagnose and remains under-recognized. The Aβ classification system differentiates patients based on the presence or absence of autoantibodies and the presence or absence of β-cell functional reserve. We suspect this patient’s presentation is most consistent with the A-β+ phenotype. As indicated by this case, the most challenging management often occurs in the subacute period when β-cell function is unknown. This case underscores the importance of recognizing ketosis-prone diabetes as a distinct clinical entity and highlights the value of close, multidisciplinary team follow-up for chronic management.

## Introduction

Atypical presentations of diabetes mellitus (DM) have been recognized in medical literature since the late 1900s. Ketosis-prone diabetes (KPD) is a unique clinical entity that does not fit the classic phenotypes of type 1 diabetes mellitus (T1DM) or type 2 diabetes mellitus (T2DM) [1-3]. KPD is defined as a syndrome in which patients with negative antibodies present with diabetic ketoacidosis (DKA), severe hyperglycemia, or ketosis due to severe β-cell dysfunction resulting in new insulin requirements [1,2]. Due to the heterogeneity of KPD, it can be extremely challenging to diagnose at the time of presentation, leading to delayed recognition. In particular, young adults presenting with hyperglycemia or DKA may be suspected to have type 2 diabetes mellitus (T2DM) or type 1 diabetes mellitus (T1DM), respectively [1]. KPD is more common among patients who are male, overweight or obese, and have a family history of T2DM [1,4]. Despite initial reports of a higher incidence among patients of African descent, KPD has now been reported as an emerging clinical entity in populations worldwide [1]. The clinical course is variable for patients diagnosed with KPD. Utilizing the Aβ classification system to differentiate KPD subtypes can provide important prognostic and management guidelines [2,3,5]. Some patients may only require high-dose insulin therapy in the acute phase, while others may require lifelong insulin administration [1]. A subset of patients will have reversible β-cell dysfunction, leading to sustained glycemic improvement and insulin independence over weeks to months [1-3]. While there are proposed metabolic mechanisms for the pathophysiology of KPD and associated β-cell dysfunction, many questions remain. We present the case of a patient initially diagnosed with T2DM who required high doses of insulin prior to a period of sustained insulin remission.

This article was previously presented as a poster at the 2026 Endocrine Society Annual Meeting on June 14, 2026.

## Case presentation

A 22-year-old man with no past medical history presented to the emergency department (ED) with one week of polyuria, polydipsia, and blurred vision. He denied any fevers, recent sick contacts, hematuria, or dysuria. No changes in appetite or bowel habits were reported. Initial vital signs showed a blood pressure of 146/83 mmHg, heart rate of 69 beats per minute, respiratory rate of 19, temperature of 36.6 degrees Celsius, and an oxygen saturation of 98% on room air. Weight was recorded as 119 kg with a body mass index of 35.5 kg/m^2^. Physical examination was notable for dry mucous membranes but otherwise unremarkable. The patient reported an extensive family history of T2DM and admitted to a recent period of heavy alcohol intake prior to admission. He did not take any prescription medications.

Laboratory values at the time of presentation showed hyperglycemia with a blood glucose level (BGL) of 421 mg/dL (SI: 23.40 mmol/L) (reference range: 74-99 mg/dL (SI: 4.12-5.49 mmol/L)) and no evidence of ketosis. The metabolic panel revealed a bicarbonate of 26 mmol/L, anion gap of 9 mmol/L, and lactate of 1.1 mmol/L. Venous pH was 7.42. Urinalysis found glucosuria (>1000) and trace ketones (Table 1). The patient was admitted for intravenous (IV) fluids and initiation of basal-bolus insulin. Hyperglycemia improved throughout admission. Hemoglobin A1c (HbA1c) could not be measured due to an unidentified hemoglobin variant, but the patient’s fructosamine level was elevated at 451 µmol/L (reference range: 200-285 µmol/L), corresponding to an estimated HbA1c of 11%-12%. At discharge, aspart 6 units with meals, glargine 20 units daily, and metformin 500 mg twice daily were prescribed. He was given a presumed diagnosis of T2DM on discharge due to his strong family history.

**Table 1:** Initial laboratory data at the time of presentation to the emergency department

Parameters	Measured value (SI)	Reference range (SI)
Metabolic panel		
Sodium	130 mEq/L (130 mmol/L)	136-145 mEq/L (136-145 mmol/L)
Potassium	4.0 mEq/L (4.0 mmol/L)	3.5-5.0 mEq/L (3.5-5.0 mmol/L)
Chloride	95 mEq/L (95 mmol/L)	98-107 mEq/L (98-106 mmol/L)
Bicarbonate level	26 mEq/L (26 mmol/L)	22-29 mEq/L (22-29 mmol/L)
Creatinine	1.11 mg/dL (97.24 µmol/L)	0.5-1.20 mg/dL (44.21-106.10 µmol/L)
Blood urea nitrogen	17 mg/dL (6.07 mmol/L)	8-21 mg/dL (2.86-7.50 mmol/L)
Glucose	421 mg/dL (23.40 mmol/L)	74-99 mg/dL (4.12-5.49 mmol/L)
Lactate	1.1 mmol/L	0.5-2.0 mmol/L
Anion gap	9 mmol/L	4-12 mmol/L
Calcium	9.9 mg/dL (2.48 mmol/L)	8.5-10.5 mg/dL (2.1-2.6 mmol/L)
Magnesium	1.7 mg/dL (0.70 mmol/L)	1.7-2.4 mg/dL (0.70-0.99 mmol/L)
Venous blood gas		
pH	7.42	7.32-7.42
pCO2	40.4 mmHg	41-51 mmHg
pO2	78 mmHg	30-40 mmHg
HCO3	26.4 mmol/L	23-27 mmol/L
O2 saturation venous	96%	40%-70%
Complete blood count		
Hemoglobin	15.2 g/dL (152 g/L)	11.7-15.7 g/dL (117-157 g/L)
Hematocrit	43.3% (0.43 L/L)	35%-47% (0.35-0.47 L/L)
White blood cell count	9,200 × 10^3^/µL (9.2 × 10^9^/L)	3,500-11,000 × 10^3^/µL (3.5-11.0 × 10^9^/L)
Platelets	301,000 × 10^3^/µL (301 × 10^9^/L)	150,000-400,000 × 10^3^/µL (150-400 × 10^9^/L)
Urinalysis		
Urine specific gravity	>1.035	1.005-1.030
Urine glucose	>1,000 mg/dL	Normal: negative
Urine ketones	Trace	Normal: negative
Urine blood	Negative	Normal: negative
Urine leukocytes	Negative	Normal: negative
Urine nitrites	Negative	Normal: negative
Endocrinology		
Thyroid-stimulating hormone	0.83 mIU/L	0.27-4.2 mIU/L
Fructosamine	451 µmol/L	200-285 µmol/L

After discharge, the patient had close follow-up with his primary care physician (PCP) and an outpatient pharmacist. At his initial PCP visit, home glucose logs showed persistent hyperglycemia. Therefore, glargine was increased to 30 units nightly and aspart to 10 units with meals. A continuous glucose monitor (CGM) was prescribed. At this time, a glutamic acid decarboxylase 65 (GAD65) antibody concentration was insignificantly positive at 0.05 nmol/L (laboratory reference range: ≤0.02 nmol/L). A hemoglobin electrophoresis was ordered to investigate his hemoglobin variant, which returned normal. The case was discussed with hematology, who recommended no additional workup given his hemoglobin level was normal and no known syndromes were identified on electrophoresis.

Two weeks later, a repeat fructosamine level was 640 µmol/L, corresponding to an estimated HbA1c of 12.49%. Basal insulin was further increased to 40 units nightly, while prandial insulin dosing remained unchanged. The patient self-discontinued his metformin due to concerns of possible side effects.

**Figure 1:**
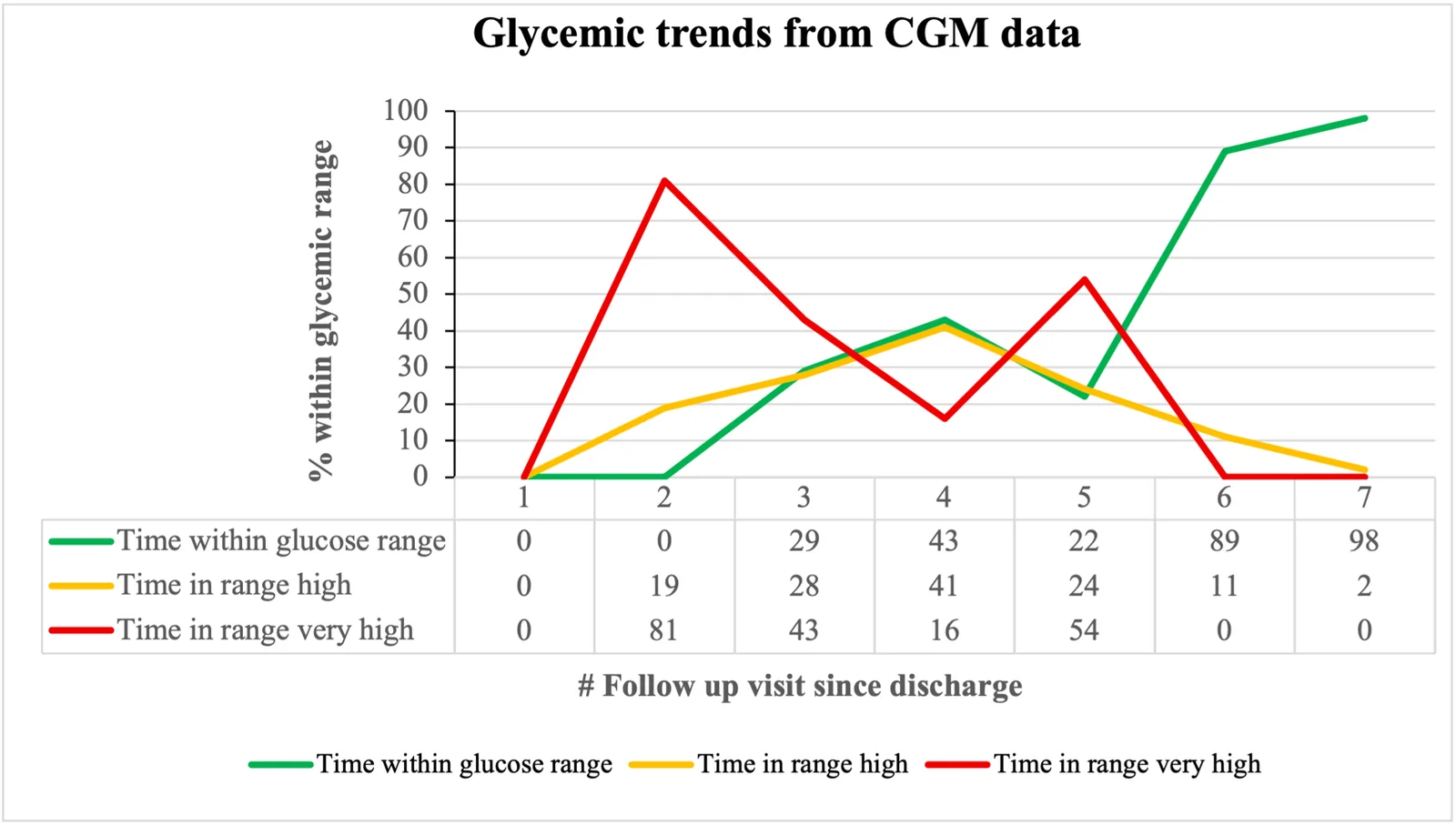
Graph depicting CGM readings from each follow-up visit after hospital discharge Time in each glycemic range is displayed for each visit. Each follow-up visit occurred at approximately two- to four-week intervals. CGM: continuous glucose monitor

**Figure 2:**
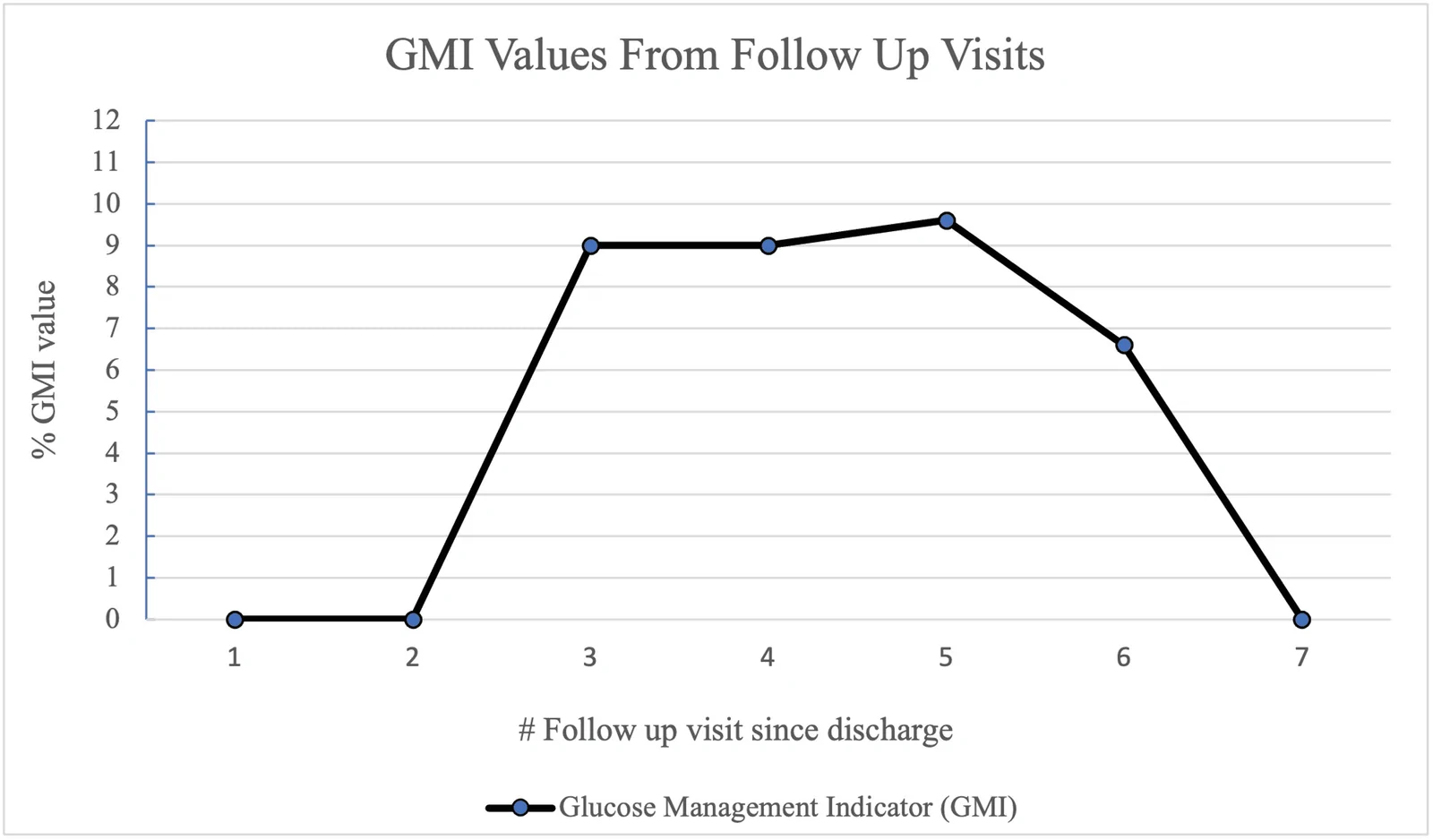
GMI values from CGM data from follow-up visits after hospital discharge No GMI data is available for visits 1, 3, and 7. The patient did not have a CGM at visit 1. For visits 2 and 7, no GMI value was displayed on the CGM report. GMI: glucose management indicator, CGM: continuous glucose monitor

The patient was seen for frequent follow-up visits, approximately every two weeks for insulin adjustment and glycemic monitoring (Figure 1 and Figure 2). Over the course of several visits, CGM data demonstrated persistent hyperglycemia (time in range: high, 19%; very high, 81%). Basal and bolus insulin were progressively increased as the patient continued to intermittently endorse polydipsia and polyuria. Glargine was titrated to 56 units nightly, and prandial aspart was maintained at 12 units. The patient reported two asymptomatic hypoglycemic events for which his CGM alarmed. He admitted missing several doses of insulin after these events due to concerns of hypoglycemia. CGM data reviewed in the following weeks demonstrated worsening hyperglycemia, and glargine was increased to 60 units nightly.

Several months elapsed following discharge before the patient could be scheduled for an endocrinology appointment. In the weeks surrounding this visit, the patient experienced symptomatic hypoglycemia with glucose readings as low as 50 mg/dL (SI: 2.78 mmol/L), prompting him to independently reduce his insulin dosage. CGM data at the time of endocrine evaluation showed marked improvements in glycemic control, with 89% time in range and 11% time in high glycemic range. All insulin therapy was discontinued due to persistent hypoglycemia, and glycemic improvement was sustained. Although C-peptide and repeat antibody testing were ordered, financial limitations prevented completion of these studies. CGM data assessed three weeks after cessation of all insulin demonstrated 98% time in range and 2% time in high glycemic range, with no hypoglycemic episodes observed. Throughout the patient’s clinical course, other differential diagnoses were considered, such as latent autoimmune diabetes in adults (LADA) or fulminant T1DM. However, his presentation of severe hyperglycemia requiring high-dose insulin treatment and subsequent remission does not align with the typical presentations of such diagnoses.

**Figure 3:**
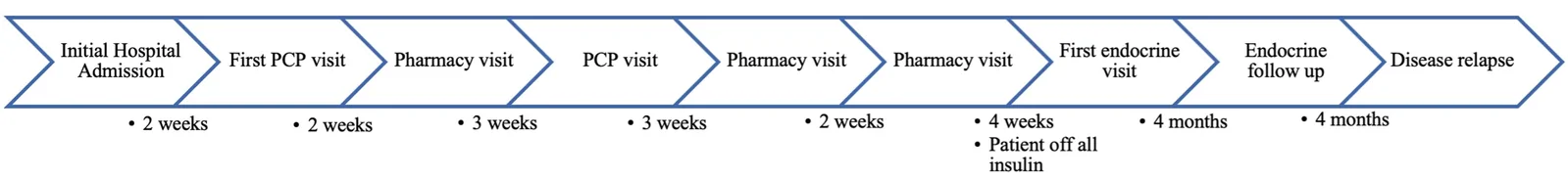
Brief timeline of events The time between each visit is indicated below the visit description. PCP: primary care physician

After approximately an eight-month period of euglycemia without insulin therapy (Figure 3), the patient presented to the hospital with symptoms of blurry vision, polyuria, and hyperglycemia identified on home finger stick measures. In the ED, his BGL was 519 mg/dL (SI: 28.83 mmol/L) with a normal beta-hydroxybutyrate (BHB) of 0.12 mmol/L (reference range: 0.6 mmol/L) and normal serum electrolytes and renal function. Urine analysis was notable for glucosuria. He was treated with 5 units of glargine and 4 units of aspart and monitored for several hours. Several days after discharge, the patient had a follow-up visit with his PCP and was restarted on metformin 500 mg twice daily and glargine 10 units nightly. The patient is pending an upcoming endocrinology appointment at this time. In the interim, the patient was adherent to all medications. He did not have any significant changes in weight and was counseled on lifestyle modifications, including routine exercise and dietary modifications.

## Discussion

This report describes the case of a 22-year-old man who initially presented with severe hyperglycemia necessitating high-dose insulin therapy for suspected new-onset T2DM. Over subsequent months, the patient experienced hypoglycemic episodes that required discontinuation of insulin. He remained euglycemic without insulin therapy for approximately eight months before an unprovoked disease relapse. As evidenced by this case, KPD is challenging to diagnose and remains under-recognized, especially in patients presenting with hyperglycemia and negative autoimmune markers [1].

There are several different classification schematics that attempt to clinically differentiate patients diagnosed with KPD [2]. The Aβ classification system provides valuable diagnostic and prognostic guidelines for management [2,3,5]. This system differentiates patients with KPD based on the presence or absence of autoantibodies and the presence or absence of β-cell functional reserve [2,4]. It is worth noting that although A+β- and A-β- patients are immunologically and genetically distinct, they share more clinical features of T1DM due to reduced β-cell function [2,4]. A+β+ and A-β+ patients are also distinct disease entities but share features of T2DM due to preserved β-cell reserve [2,4]. In this case, the patient lacked significant autoimmune markers and demonstrated β-cell functional reserve. His GAD65 antibody concentration measured 0.05 nmol/L, a value regarded as clinically insignificant due to minimal elevation (normal: ≤0.02 nmol/L). The patient’s progression from severe hyperglycemia necessitating high-dose insulin therapy to subsequent insulin independence aligns with the A-β+ classification. β-cell function can typically be assessed by measuring C-peptide levels; unfortunately, this patient’s insurance would not cover C-peptide testing or repeat antibody testing. Repeat laboratory evaluation would have further supported differentiating the subtype of KPD based on the Aβ classification system and provided information regarding β-cell function. We recognize that lack of repeat testing is a limitation in order to definitively classify the patient’s type of KPD. However, the rapid de-escalation of insulin requirements in this patient likely indicated sufficient β-cell functional reserve and contributed to his period of remission. Patients with A-β+ KPD demonstrate significant β-cell recovery and often sustained insulin independence [2,3,6]. Close monitoring is essential as relapse can occur unprovoked or during periods of physiologic stress [3]. In particular, a large portion of patients with A-β+ KPD tend to experience unprovoked episodes of DKA or hyperglycemia [6].

The exact pathophysiology of KPD remains unknown but is hypothesized to occur in susceptible patients due to defective energy production from impaired ketone oxidation and fatty acid utilization [3,7,8]. In patients diagnosed with A-β+ KPD, data suggest that severe hyperglycemia and glucotoxicity effects may blunt intracellular pathways, causing decreased insulin secretion and oxidative stress in β cells contributing to the characteristic reversible β-cell dysfunction [3,7-9]. Another potential consequence of glucose toxicity is downregulation of skeletal muscle insulin signaling, which may also exacerbate β-cell dysfunction [3,9].

Management of KPD largely depends on the clinical setting and an individual patient’s clinical course. Acute presentations of hyperglycemia, DKA, and electrolyte abnormalities should be treated according to established protocols. Possibly the most variable and challenging management occurs in the subacute period when β-cell function and reserve are unknown. It is imperative that insulin dosing and blood glucose levels are monitored closely, preferably with a CGM, as consequences can be fatal. Due to β-cell dysfunction, insulin requirements tend to be higher in the first two to four weeks after acute metabolic decompensation [2,10]. Insulin requirements afterward may progressively decrease as β-cell function recovers [4,5]. Close monitoring and classification are essential to prevent unnecessary insulin administration and hypoglycemia, as recovery is variable. Vigilant monitoring of symptoms and BGL will be a lifelong challenge for many patients because of the unpredictable and unprovoked relapses in hyperglycemia that can occur, which is evidenced in this case [6]. Patient education and close follow-up with outpatient primary care, pharmacy, and endocrinology are extremely valuable.

## Conclusions

This case underscores the importance of recognizing KPD as a distinct clinical entity to ensure accurate classification and management. It is important that all clinicians maintain a broad differential diagnosis for patients with atypical presentations of diabetes mellitus. Importantly, this case highlights the value of close, multidisciplinary follow-up. Careful outpatient monitoring allows for timely adjustment of therapy to mitigate the risks associated with both hyperglycemia and insulin treatment during the subacute period. Following acute presentation of hyperglycemia or ketoacidosis, precautionary measures such as patient education and prescriptions for glycemic monitoring supplies are essential for patient safety.
